# Using Blockchain Technology to Mitigate Challenges in Service Access for the Homeless and Data Exchange Between Providers: Qualitative Study

**DOI:** 10.2196/16887

**Published:** 2020-06-04

**Authors:** Anjum Khurshid, Vivian Rajeswaren, Steven Andrews

**Affiliations:** 1 Dell Medical School The University of Texas at Austin Austin, TX United States; 2 University of Colorado at Denver Anshutz Medical Campus Aurora, CO United States

**Keywords:** blockchain, distributed ledger technology, health care, data sharing, homeless, data autonomy

## Abstract

**Background:**

In the homeless population, barriers to housing and supportive services include a lack of control or access to data. Disparate data formats and storage across multiple organizations hinder up-to-date intersystem access to records and a unified view of an individual’s health and documentation history. The utility of blockchain to solve interoperability in health care is supported in recent literature, but the technology has yet to be tested in real-life conditions encompassing the complex regulatory standards in the health sector.

**Objective:**

This study aimed to test the feasibility and performance of a blockchain system in a homeless community to securely store and share data across a system of providers in the health care ecosystem.

**Methods:**

We performed a series of platform demonstrations and open-ended qualitative feedback interviews to determine the key needs and barriers to user and stakeholder adoption. Account creation and data transactions promoting organizational efficiency and improved health outcomes in this population were tested with homeless users and service providers.

**Results:**

Persons experiencing homelessness and care organizations could successfully create accounts, grant and revoke data sharing permissions, and transmit documents across a distributed network of providers. However, there were issues regarding the security of shared data, user experience and adoption, and organizational preparedness for service providers as end users. We tested a set of assumptions related to these problems within the project time frame and contractual obligations with an existing blockchain-based platform.

**Conclusions:**

Blockchain technology provides decentralized data sharing, validation, immutability, traceability, and integration. These core features enable a secure system for the management and distribution of sensitive information. This study presents a concrete evaluation of the effectiveness of blockchain through an existing platform while revealing limitations from the perspectives of user adoption, cost-effectiveness, scalability, and regulatory frameworks.

## Introduction

### Background

According to Austin’s Ending Community Homelessness Coalition (ECHO), more than 7100 people in Austin experienced homelessness and needed housing and other supportive services in 2016 [1]. One of the many barriers to housing for persons experiencing homelessness (PEH) is the lack of access to their own documentation and the time and money required to replace lost or stolen documents. Client data are often distributed between service providers, causing a deterioration in health and delays in service when accessing data from multiple sources. Existing database systems such as Homeless Management Information System (HMIS) to manage documentation for the PEH lack interoperability for different document formats and organizations outside the system, do not facilitate data ownership for clients, and cannot guarantee data privacy and security.

The Mayor’s Challenge Competition, sponsored by Bloomberg Philanthropies, is designed to facilitate innovative and scalable solutions for city leaders to tackle urgent local challenges. Austin, Texas, was one of the 35 cities selected to prototype and test their ideas over 6 months with a grant support of US $100,000. Through a partnership between Dell Medical School, the City of Austin government, Austin Travis County Emergency Medical Services, and community organizations, a pilot study was conducted to test the first use of blockchain technology to validate identities and improve access to services for the PEH in the country.

### Objectives

To solve these problems of *transaction identity*, we previously proposed the use of distributed ledger technology (DLT), or blockchain technology, to provide a validated, immutable identity and record of service transactions [2]. The literature shows promise using blockchain for data control in electronic health records, public health surveillance, disease management, genomic and biomedical data, and clinical trials [3-9]. Blockchain is currently used in rural Indonesia, Syrian refugee camps, and the slums of Kenya for similar identity management applications. Key life events such as birth registration, immunizations, health provider visits, and agricultural harvests are securely recorded and maintained on a blockchain, creating an immutable, accessible, and portable identity for displaced and marginalized people. Following this model, we explored the use of a true *economic passport* for the homeless, working toward the goal of ending homelessness. This technology was chosen as a solution over other methods of storing and sharing information such as a digital wallet or a relational database because its features met the requirements of our use case. We have continued this study, and here, we provide results from our testing of the deployment of this approach.

Blockchain is a technology for shared databases used by multiple writers in which each organization’s entries must be verified and cannot be modified by an outside party. When transactions from one entity are needed by another to provide a service or verify a document, storing transactions in a single shared database promotes expedient exchange of information. A regular shared database can reliably manage the permissioning of multiple authorized users to view data. However, a relational database system in which various entities update and write information can result in unrecoverable errors. Maintenance of a health and social service information system involves backup storage services, recovery mechanisms, and updating information [7]. In the event of a database server failure, the entire system is affected, and information can be lost if it has not been backed up and stored. Ownership of the master file by a centralized user also accords control to a single party; thus, there is no inherent safeguard against data tampering, and the integrity of data cannot be guaranteed [10].

Blockchains organize data so that secure transactions are approved and recorded through consensus from entities on the chain [10,11], providing greater error checking and transactional validity than relational shared databases [10-12]. A blockchain distributes data across the network, with data copied on each node of the chain [13]. Each node installs the genesis block or the first block in the chain [10]. A group of validated transactions is added to a new block with the file attachment, sender, receiver, timestamp, and cryptographic hash of the previous block [14]. A hash is a one-way encryption function, which is used to generate a public and private key for each user [15]. Information sent over the blockchain is secured by a user’s private key and cannot be viewed or modified without the key [12,13]. Data are encrypted and unintelligible to protect private information stored on the blockchain or in the event of a security breach [14]. An individual block’s hash depends on the hash of the previous block, locking transactions together [12,14]. Modifying data in one block would alter all subsequent blocks, making the blockchain an immutable and authoritative record of transactions [10,12-14]. Blockchain also uses a consensus validation mechanism replacing a trusted third-party intermediary or a manual offline reconciliation process with peer-to-peer protocols, allowing organizations to agree upon submitted entries without a singular point of failure or control [10-12,16]. A network of users collectively adheres to previously agreed upon rules automatically implemented to verify the authenticity of transactions and ordering of records added to the chain [12,16].

A *public* blockchain is completely decentralized, and transactions depend on consensus from a majority of nodes. In a *private* blockchain, users are granted access by permission from the owner of the blockchain [10]. For sensitive data such as personal identity records, private blockchains limit transaction visibility to authorized users and promote scalability because of greater user and transactional control [12]. A private blockchain is only partially decentralized because nodes are limited to trusted users with varying degrees of access and sharing permissions. If a conflict or security breach arises, the system can be recovered from any user and timestamp [10]. The only security issue is that a majority of nodes could collude to rewrite the chain, as there is a partially centralized authority controlling the nodes in the chain. However, in a permissioned system, it is unlikely that users aim to violate the immutability of the blockchain, as they are trusted entities using the blockchain for organizational or personal benefit [12,16-18].

Lack of interoperability between service providers arises from nonuniform data formats and storage methods [13]. Blockchain creates an accessible and authoritative ledger of diverse document types, acting as a method for storing and gathering information from multiple independent systems [6]. Off-blockchain data storage can be integrated for large files or extensive data storage in a variety of formats and is scalable to include a larger health and social service ecosystem. The blockchain can also be used to facilitate communication for application programming interfaces (APIs), which can restructure, aggregate, and merge data from various sources in a standardized format. A blockchain-based API model allows for decentralized and authoritative data exchange between systems, user identification, a validated transaction history, and proof of transaction legitimacy [19].

In this study, we describe the results of a pilot test using a blockchain solution to mitigate the current challenges in service access for the homeless and data exchange between providers. The potential for blockchain in various health care settings has been examined in several studies [3,4,6-9,14,20,21]. However, these studies are theoretical in nature, and to our knowledge, no published studies have examined the feasibility, effectiveness, or performance of blockchain in a real-life public setting. In this study, we used a private, permissioned blockchain system for secure storage and transmission of documents with planned API integration for intersystem data access and transmission from legacy databases. This model additionally permits individuals to access and control their own data by uploading official documents and sharing self- or provider-uploaded records with select organizations. Our approach in this study aimed to increase agency, motivation, and control while reducing service barriers for the homeless and supplying more complete and accurate information for service providers.

## Methods

### Generating Testable Assumptions

Austin, Texas, was 1 of the 35 cities selected by the 2018 Mayor’s Challenge Competition, sponsored by Bloomberg Philanthropies. The competition was designed to facilitate innovative and scalable solutions for city leaders to tackle urgent local challenges. Each Champion City selected in the Mayor’s Challenge Competition was to prototype and test their ideas over 6 months. Through a partnership between Dell Medical School, the City of Austin’s Office of Technology Innovation, Austin Travis County Emergency Medical Services, and community organizations, a pilot study was conducted to test the use of blockchain technology to validate identities for the homeless.

Participation in the Mayor’s Challenge Competition required a series of steps to design and implement testing. First, we identified assumptions underlying the idea, which will be required for stakeholder participation. We found that the following set of assumptions must be tested and shown to be valid for the successful implementation of our solution (see Textbox 1): (1) our prototype platform would be appropriate for PEH in Austin, (2) PEH will be able to understand its features and will consent to participate in such a system, (3) providers will be able to access and trust the information on the platform, (4) blockchain technology is essential to manage identity for PEH, (5) privacy and confidentiality of data will be protected, (6) service delivery will be facilitated, and (7) interorganizational efficiency will increase by sharing data in a standardized manner with shared governance on the platform. After determining how to test and evaluate these assumptions, we developed and modified prototypes and tests based on real-life findings, user feedback, and an evolving understanding of our idea. For prototype testing, we used an existing blockchain technology platform that had been implemented outside the United States to manage the identities of refugees.

Second, we identified markers for what constitutes an appropriate test of a platform based on blockchain technology (see Table 1). To be effective, the platform needs to be able to allow individuals to enter the system by creating an account. They need to be able to upload documents and then share those documents with others, with control over which documents get shared with which other participants. Service providers need to be able to additionally conduct transactions, especially transactions of identity validation, and to share information with each other. All these actions and pieces of information need to be recorded and available, to be differentially accessible based on user-controlled permissions, and to remain securely protected and immutable. The system overall needs to be easily understood by users.

Before testing our assumptions, initial engagement with the homeless population was performed to determine their needs and concerns. Two-hour meetings were held biweekly over a period of 12 months. Attendees included 20 homeless individuals in the City of Austin and 1 to 2 staff members from the City of Austin Office of Innovation who regularly interacted with the homeless population. The homeless participants were rotated monthly to include a larger subset of the population, and each participant was compensated for their time at a rate of US $20 per hour. Meetings consisted of open-ended questions regarding difficulties those with lived experience of homelessness face in daily life, interacting with service providers, maintaining documents, and filling out applications.

**Figure 1:**
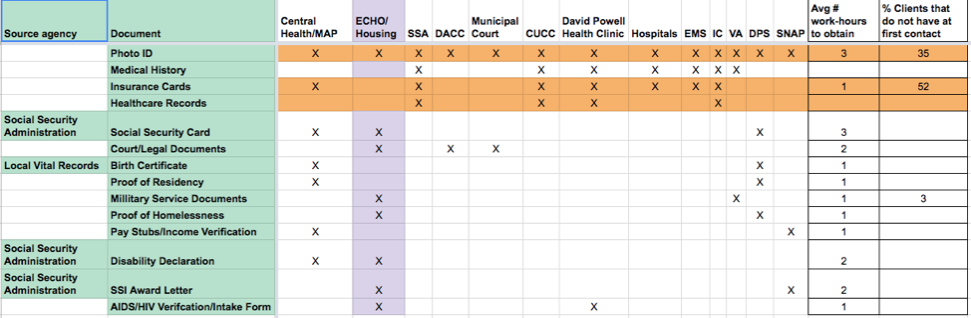
Documents needed across service providers and their homeless clients. CUCC: Community Care Clinics; DACC: Downtown Austin Community Court ; DPS: Department of Public Safety; ECHO: Ending Community Homelessness Coalition; EMS: Emergency Medical Services; IC: Integral Care; SNAP: Supplemental Nutrition Assistance Program; SSA: Social Security Administration; VA: Veterans’ Administration.

On the basis of these discussions and the answers we received to the questions in Table 2, we developed a list of documents that are most useful for testing our solution. Figure 1 lists types of documents and types of organizations and gives a sense of how difficult it is to reobtain a particular document. The figure provides insight into what a person experiencing homelessness needs to be able to manage to prove their identity to receive services, especially those that provide health care and potential housing.

As can be seen, the documents required for identity verification varied across types of organizations. Every organization, though, needed an official photo ID; yet, about one-third of clients lacked such a basic identity document when first entering the system. Replacing a photo ID is a time-intensive effort. Approximately half of the presenting users lacked insurance cards, which were required by many organizations. Looking at the entirety of the figure, though, it becomes clear that managing identity involves managing a significant number of identity documents across a wide audience of service organizations.

As some of the assumptions had overlapping aspects, we describe our methodology in testing all 7 assumptions in the subgroups below.

**Testable assumptions for solution implementation.**

1. Given that our blockchain platform is modified from a pre-existing platform for refugees, the use case of refugees must be nominally equivalent to the use case of people experiencing homelessness
2. To gain user participation, people experiencing homelessness must understand the functionality and features of the platform, and consent to participate.
3. Stakeholder participation requires providers to access, accept, and trust information shared through the blockchain network.
4. Blockchain is necessary to meet our goals.
5. Privacy and confidentiality of personal data needs to be protected.
6. Enabling access to information facilitates service transactions.
7. The technology enables a shared data standard and governance that increases inter-organizational efficiency.

**Table 1:** Blockchain components needed for a successful prototype.

Test	Elements needed
(1) Allow individuals to create accounts and upload documents	Profiles Ability to connect profiles Individual document repository Ability to grant repository and asset level access
(2) Allow individuals to share documents with differentiated permissions	Ability to find other profiles in the system Ability to send a document Ability to share a document Profile user-controlled permission Data level permission
(3) Allow service providers to share documents and conduct transactions, including verification of service use	Ability to see a validated document Ability to share a document Ability to see permissions by individuals
(4) Allow service providers to share with each other	Ability to find other verifiable users/profiles
(5) Capture all transactions/records	Ledger with varied permissions All actions write to a ledger Inability to delete from ledger

**Table 2:** Research questions.

Particpants	Questions
For persons experiencing homelessness:	Which things on your to-do list frustrate you the most? Which records or documents are you in most need of accessing? If you could reduce a barrier to a daily goal, what would it be?
For community health paramedics, when you help the homeless:	Where do you see the most missed opportunities? What common miscommunications could be prevented if you had direct information or document access? What information is lacking that would help you the most?

### Testing Assumptions 1 and 5: Usability and Security of the Platform

Assumptions 1 and 5 regarding the viability of an existing private, permissioned blockchain platform for our use case and the security and privacy of data were tested in 2 sessions on June 14, 2018, and July 13, 2018. In the first session, our testing plan was for the homeless clients to set up an account, upload a document onto the blockchain, deny a request to connect from an organization, accept a request to connect from an organization, and participate in a 3-party transaction between the client and 2 service providers. All tests were performed with platform provider staff, our team members, and 2 homeless individuals. Members of our team created mock provider accounts before testing with our homeless participants. For our second testing session, we modified the user interface so that an account profile contained the user’s picture and name with categories for general profile information, identity documents, medical documents, and dependents or emergency contacts.

### Testing Assumption 2: User Participation and Understanding of the Technology

We tested our assumption regarding user participation (assumption 2) at 2 pop-up resource clinics, where the homeless could access multiple health, social, and other service interventions. Our group of 8 city officials and 5 individuals from our team engaged a total of 34 homeless residents. Of the 34 homeless residents, 15 agreed to participate, 11 indicated interest when more information and a working platform were available, and 8 either declined to participate or did not finish the discussion.

**Figure 2:**
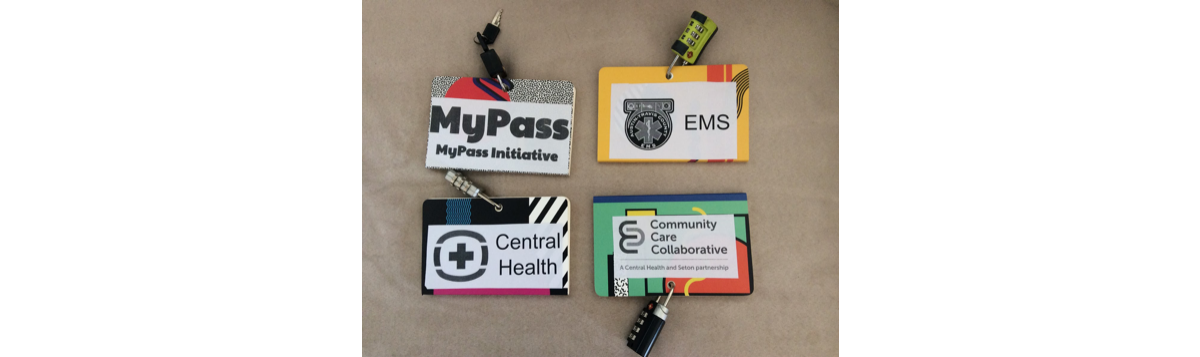
Distributed ledger technology prototype.

Attendees were offered the opportunity to discuss and provide feedback on a technology designed to securely store and share their documents. In our discussions with homeless participants, we used 3 different prototypes of our platform with increasing levels of fidelity to a real-life DLT platform. At the lowest level, to explain the concept of DLT in a nontechnical manner, we created a prototype of the platform using several journals with a lock and key (Figure 2). Each journal represented the record of a user or a service provider. In a DLT, when a document (or other record) is written in the journal or uploaded onto the blockchain, none of the other users in the individual’s network can see its contents until permission is granted, even if they are aware that there is an existing entry in the ledger (or block on the chain). In our testing analogy, granting permission is represented by the key that opens the lock to a specific physical journal. We also demonstrated distributed data storage by tearing up a piece of paper from a user’s journal and spreading the pieces into buckets representing different institutions. In this scenario, when a document was accessed with permission, the pieces were reassembled.

Increasing the fidelity of the prototypes, we next showed screenshots of the platform prototype with sample transactions, and then, at the highest fidelity level, a digital prototype on a mobile phone with uploaded data was presented. These 2 prototypes were used to further explain the technology and platform concepts. The static screenshot-based prototype showed prospective categories (eg, identity, residency, medical profile, employment history, education, and children/preferred contacts) for the different types of documents. We also showed screenshots of a request from a provider for permission to see a user’s data, accepting and giving the provider permission to view specific documents, and a transaction history showing that a document was shared with a provider.

At this stage in our project, the high-fidelity prototype platform was then used to demonstrate uploading and viewing a picture of a driver’s license via the platform. The prototype platform for refugees had not been modified at all for our use case. Thus, our demonstration was performed in a sandbox or in a closed, nonlive testing environment to safely experiment with Web or software projects. We additionally decided to conduct further tests with sample documents until the technology was fully configured and free of errors.

Understanding and acceptance of metrics were measured with participation consent forms and qualitative feedback through open-ended questions.

### Testing Assumptions 3, 6, and 7: Stakeholder Participation and Organizational/Data Standards

We tested assumptions 3, 6, and 7 regarding stakeholder participation and organizational and data standards through interview questions with representatives of the Downtown Austin Community Court, Central Health, and ECHO who interact with and provide services to PEH regularly (Multimedia Appendix 1). The participants were initially explained the technology platform, its relevant features, and our assumptions to test its practicality in addressing PEH identity management for health care and social services. Each representative was provided an opportunity to respond to the semistructured, facilitated discussion.

## Results

### Test of Assumptions

As was clear in the Methods section, multiple assumptions were tested in each of the approaches. As such, we note which assumption(s) was being tested, where appropriate. In particular, note that assumption 6 about data availability facilitating service transactions permeates most aspects of the testing process. The assumptions are listed in Textbox 1, and more detailed testing results are listed in Multimedia Appendix 2.

### Assumptions 1 and 5

In testing assumptions 1 and 5, both of our homeless participants were able to set up a user account, accept a connection request, deny and accept a provider connection request, and exchange transactions to and from providers. Specific observations, difficulties encountered, and their relevance to our project and testing assumptions are displayed in Multimedia Appendices 2 and 3. During the second session, with modifications to the test, some of the onboarding workflow resulting from entering extensive personal information created a more streamlined user experience in terms of finding and categorizing types of documents. However, testers still experienced several challenges (Multimedia Appendices 2 and 3) in addition to those previously documented in Multimedia Appendix 2 regarding platform features and functionality. In general, the problems that users experience tend to fall into 5 buckets: trusting self-uploaded documents, understanding blockchain concepts, platform performance issues, confusion about the workflow, and privacy concerns. We return to this in the discussion below.

Overall, users on the platform were able to accomplish the tasks expected of them even if guidance was required, but there were definite issues that need to be solved. Trying to use an existing platform developed to assist refugees without modification did not translate to the use case of working with those experiencing homelessness. Furthermore, privacy and security did indeed turn out to be of paramount importance to end users.

### Assumptions 2 and 6

The results of our tests showed that the homeless can understand the platform and its potential benefits, and a majority of individuals consented to participate immediately or at a later date. Feedback from our PEH test participants included the need for a wider variety of documents that can be validated and authenticated on the platform, leading us to consider possibilities for future expansion, such as involving state or federal institutions. We also planned to create benchmark documents facilitating services such as housing, disability benefits, Supplemental Nutrition Assistance Program (SNAP), medical, court documents, and insurance. Each benchmark document would consist of validation from multiple providers that the documents necessary for a service have been obtained and authenticated, increasing efficiency for service providers and improving access to services for their homeless clients.

### Assumptions 3 and 6

The results showed that providers will accept data shared through the blockchain platform, but permissible document fidelity varies across organizations. Specifically, the requirements of what would be considered a certified document varied (see Table 3). Transactions requiring hard copies included employment documents, bank transactions, social security, and vital records, which would be difficult to include without altering the standards for data. The Medical Access Program Card (a local health insurance card), SNAP application documents, and housing applications permit document copies and can easily facilitate transactions without adjusting data standards.

The need for interorganizational records or documents from clients also varied. Many providers kept internal digital copies of client records and provided services to the homeless without proof of identity because of flexible policies for this population. Identification documents are unnecessary for social security, disability benefits, transitional housing, or rehabilitation services, but the lack of identification documents can delay medical care at hospitals. These providers envisioned a role for a distributed ledger system when clients did not grant permission for document storage and for time-sensitive documents such as a current utility bill needed to prove residency. In these cases, system-level efficiency could be improved if provider employees knew what type of documents were available and if documents of interest could easily be located without looking through extraneous information.

**Table 3:** Level of document certification

Certification level	Definition	Requirements to meet level of certification	Examples
Validated	Approved to be valid document and belong to the holder (platform user)	Documents coming directly (digitally and physically) from the originating Source	Birth certificate uploaded and sent by Vital Records Medical record sent by a doctor Medical Assistance Program (MAP) card sent by the issuing entity
Verified	Verified that document belongs to the holder (platform user) and document is what it claims to be	Physical document must be seen by the certifying entity	ID that is seen in person by Austin Police Department (APD) and uploaded or sent to a user
Uncertified	Exists as a document/asset, but not marked as validated or verified	None	A self-uploaded form, such as a Homelessness Statement

### Assumption 4

Potential changes in the organizational workflow to accommodate a new system, such as our platform, was another area of friction. The HMIS provided by the US Department of Housing and Urban Development (HUD) is already widely used across the country as a centralized database to confidentially aggregate data on the homeless and housing provisions provided to these individuals. Copies of documents and IDs can be stored via HMIS, and organizations funded by HUD are required to use HMIS for client data. From the service provider perspective, blockchain is most useful for documents not currently stored in the HMIS database or for client personal use to keep track of future appointments and pending documents needed for services. This showed that the blockchain platform may, for the time being, work complementary to other existing databases or information systems, adding new features that are difficult to establish using legacy systems.

### Assumptions 5 and 6

Technological concerns included the privacy and confidentiality of indelible and immutable client data for a service that might not be successful in the long term. Providers agreed that the technology showed value for the nonhomeless population as well, but opinions on whether the technological barriers were surmountable for the homeless were mixed. The most vulnerable members of the homeless population struggle to keep a phone and remember their email IDs and passwords, complicating platform access and use. There will always be a level of tension between ease of use and privacy/security concerns. However, adequately addressing the latter may help facilitate more practical solutions for the former. The readiness to overcome any specific requirements for the use of the solution by PEH seemed to be determined by how many of their problems were being solved effectively by using such a platform.

### Assumption 7

Analysis of interview feedback elucidated the requirement of clearly defined roles for each organization. Not all providers wanted responsibility or additional work processes of account creation, document validation, and resolution of transactional or account legitimacy. Legal issues and worries regarding Health Insurance Portability and Accountability Act (HIPAA) compliance to share personal health information and other client data between organizations were further constraints on stakeholder roles, participation, and interorganizational efficiency. The questions around governance and standardized processes for onboarding and managing roles remained unresolved. It was not clear whether the City of Austin had the capacity to manage a blockchain platform or if there was another organization in the city, which had the capacity and trust of all other stakeholders and general citizens, including the PEH.

### Assumptions 1 and 4

The results of our tests demonstrated that the platform we had chosen to use lacked core functionality and configuration specifically needed for our particular use case. The user experience was time consuming and not intuitive for tasks such as account creation and sharing of data, which will likely hinder client and provider understanding, acceptance, and adoption of the platform. Validated data could not be securely shared while protecting privacy and confidentiality; thus, data transactions on the blockchain platform cannot facilitate services or increase interorganizational efficiency. The nature of sharing and transacting data on this platform was specific to the original use case of small farmers and refugees and was not suitable for our purpose of securely sharing and storing documentation. As our contract with the platform provider was constrained to limited modifications because of the time frame and budget of the project, we were unable to make the necessary changes to continue testing this platform for our use case. Given these findings, testing assumption 4 on the equivalence of the 2 platform use cases was invalidated.

## Discussion

### Principal Findings

Secure, fast, and reliable sharing of validated health and identity data is crucial to improve the quality of life and health outcomes of the general population, particularly vulnerable populations such as those experiencing homelessness. Our project tested whether a blockchain-based platform had the functionality to manage permissioned access and distribution of data while empowering patients with control over their own records. This study also showed the need to address challenges in establishing and operationalizing a blockchain system before trial or full-scale production.

Although broader and timely access to health and identity records can be achieved through blockchain, there are costs in transferring to a new system and training professionals and patients on the best methods of use to improve efficiency and outcomes. In the underserved populations, initial adoption depends on a user-friendly interface and an end user experience accounting for varying levels of technological access and ability. Unfamiliarity with blockchain technology and usage also creates challenges in adoption for service providers. Understanding the basic principles of blockchain technology is necessary to confer trust in the system and allow changes in workflow, promoting organizational efficiency and preventing the burden of additional verification of authorized documentation. Institutions participating in a blockchain system must also mutually agree upon the size of data that may be stored or transferred on the blockchain to maximize system performance [7,12].

Our research showed that a blockchain can be used to manage personal and health data by facilitating interoperability, patient control of documents, and a record of consented document access while maintaining data privacy and security. A validated, immutable, and decentralized ledger promotes system and transactional trustworthiness but cannot guarantee the absence of falsifications or errors from the point of origin. These mistakes are perpetuated in the blockchain without a manual content verification procedure [10,22,23]. Our results show that a private, permission-based blockchain might be suitable for sensitive personal and health information with regulatory guidelines and standards to ensure appropriate use of data. In a permissioned system, participating organizations need to decide who is responsible for the creation of new accounts [10]. Individual users may also request for their data to be erased [11]. If the data are protected health information (PHI), HIPAA mandates its deletion in the event of unauthorized access. PHI must also be destroyed when a data storage device is decommissioned. This is only possible when documents are not stored in the blockchain. If data are stored in an off-blockchain data repository or database, a record of the existence of deleted data may still be maintained within the chain [24]. There is ambiguity regarding whether metadata of PHI are considered PHI [25], but legal counsel with respect to the application of HIPAA and data privacy standards is vital to ensure compliance with regulatory frameworks [24,26]. A potential limitation regarding these data and use standards arises with respect to a private, partially centralized blockchain. To achieve compliance and vendor neutrality, an outside enforcing authority may be required [12].

### Limitations

Timeline and budgetary constraints from the Mayor’s Challenge Project limited our ability to fully modify the platform for our use case. We were unable to develop an end user experience promoting homeless client adoption or develop changes in workflow, policy, and data sharing agreements for the best use of blockchain technology. The significant security and usability issues with our original platform prevented large-scale implementation in an empirical setting.

Further research is required to compare the efficacy and costs of an approach based on blockchain technology with other alternative approaches. Although the research presented here shows the potential of a blockchain-based approach, we need to better understand the comparative benefits and costs. For example, the City of Austin could have created a central database to which all partners and collaborators would agree to add their data. Questions on whether all participants would adopt the use of standard data types and a centralized, city-owned database would need to be addressed. Experience has shown that not all advocacy groups or people experiencing homelessness fully trust the city government, for example. The purpose here, however, was to show whether a blockchain-based approach could work, and it was not to address the question of comparative effectiveness.

### Comparison With Prior Work

Several studies to date have explored the potential applications of blockchain to solve key issues in the health care sector. This study demonstrates a methodology and rigor that may be needed to test if a blockchain can be used to securely store and track verified documentation, promote client ownership of data, and improve interoperability by facilitating permission-based data sharing. This study paves the way for future studies by detailing specific organizational, logistical, and system considerations for successful and scalable implementation.

### Conclusions

Blockchain may provide a means for consented access to validated personal and health data, thus increasing interoperability without compromising the security or privacy of data [12,24]. Existing solutions need to be put through rigorous testing before being adopted at scale. We developed assumptions based on feedback from end users, PEH, and service providers on important aspects to be tested. We engaged these groups actively to test the assumptions using a preexisting platform and found that many of the assumptions could not be validated, given the constraints of the platform, limitations on time and resources in the pilot, and lack of clarity on legal and compliance implications of this new technology. On the basis of our learnings through this pilot study, we opine that using an off-blockchain data lake or extant provider databases for PHI storage and systematic storage of an index of health records and associated metadata on the chain can permit the management of access and data control while complying with privacy and regulatory standards [24,27]. Maintaining limited personal data on the blockchain maximizes the speed of transactions and scalability of the blockchain system [11,12,20]. Through an API, organizations can integrate data and receive accurate, updated information in a usable format [19]. Faster availability of real-time data reduces delays in service [10,11,28] and promotes coordinated health care and specialized treatment based on outcomes and efficacy. Shifting data ownership and control to the individual optimizes access to health and social services and engages a patient in their own care through selective sharing of information and data with providers or researchers [29-31]. Patient-reported measurable outcomes and data from mobile apps or on-person sensors may also be integrated, creating a single access point for all real-time health data and improving personalized health care [12,13]. These benefits outweigh the challenges in adoption, employment, and investment of a blockchain system. The application of current recommendations and continued research into blockchain implementation is crucial to develop cost-effective strategies for the operationalization of blockchains while ensuring efficiency, data privacy, and scalability in the health care ecosystem [24].

## Appendix

Multimedia Appendix 1 Interview questions for service providers.

Multimedia Appendix 2 Platform testing session results.

Multimedia Appendix 3 Second session findings.

## Abbreviations

API: application programming interface
DLT: distributed ledger technology
ECHO: Ending Community Homelessness Coalition
HIPAA: Health Insurance Portability and Accountability Act
HMIS: Homeless Management Information System
HUD: US Department of Housing and Urban Development
PEH: persons experiencing homelessness
PHI: protected health information
SNAP: Supplemental Nutrition Assistance Program
