# Supplementary figures and images for: Using Blockchain Technology to Mitigate Challenges in Service Access for the Homeless and Data Exchange Between Providers: Qualitative Study
Source: J Med Internet Res. 2020 Jun 4;22(6):e16887. doi: 10.2196/16887 (PMC7303832; doi:10.2196/16887)

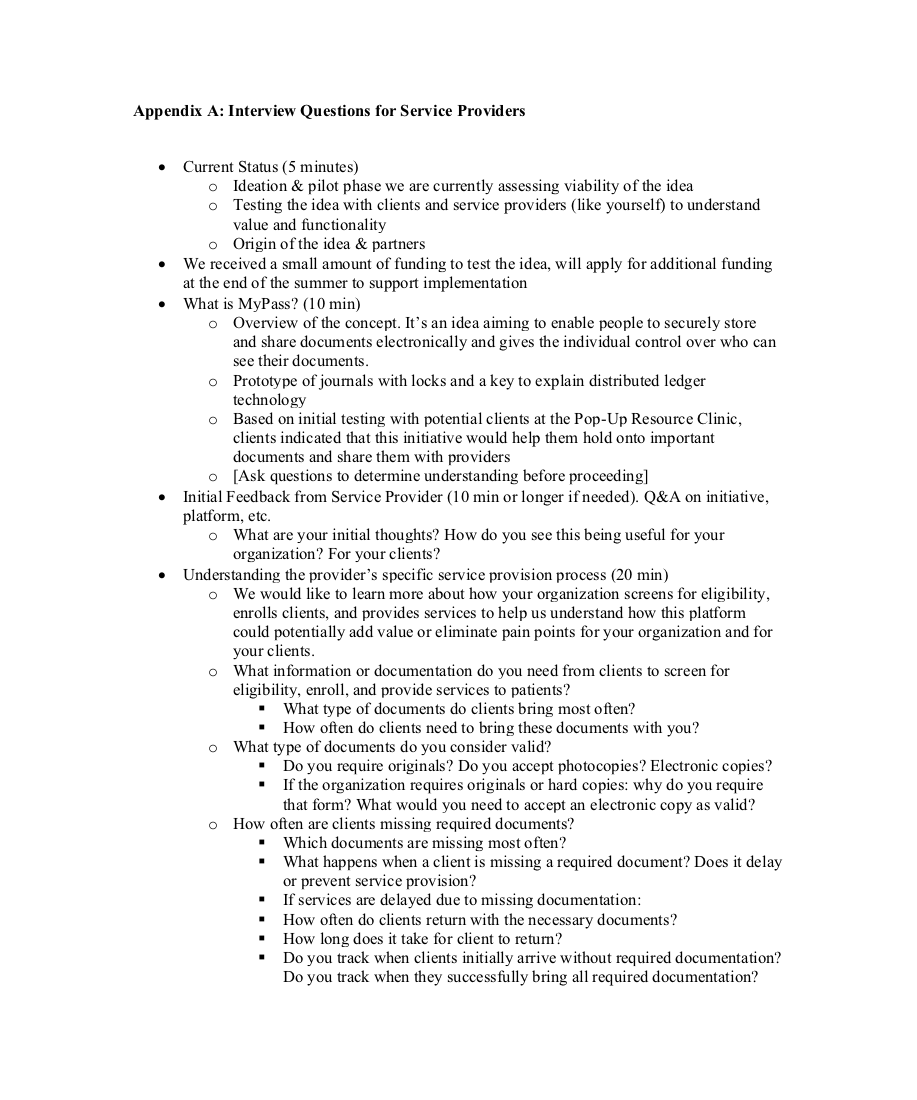

Supplement: Multimedia Appendix 1 [file jmir_v22i6e16887_app1.png]

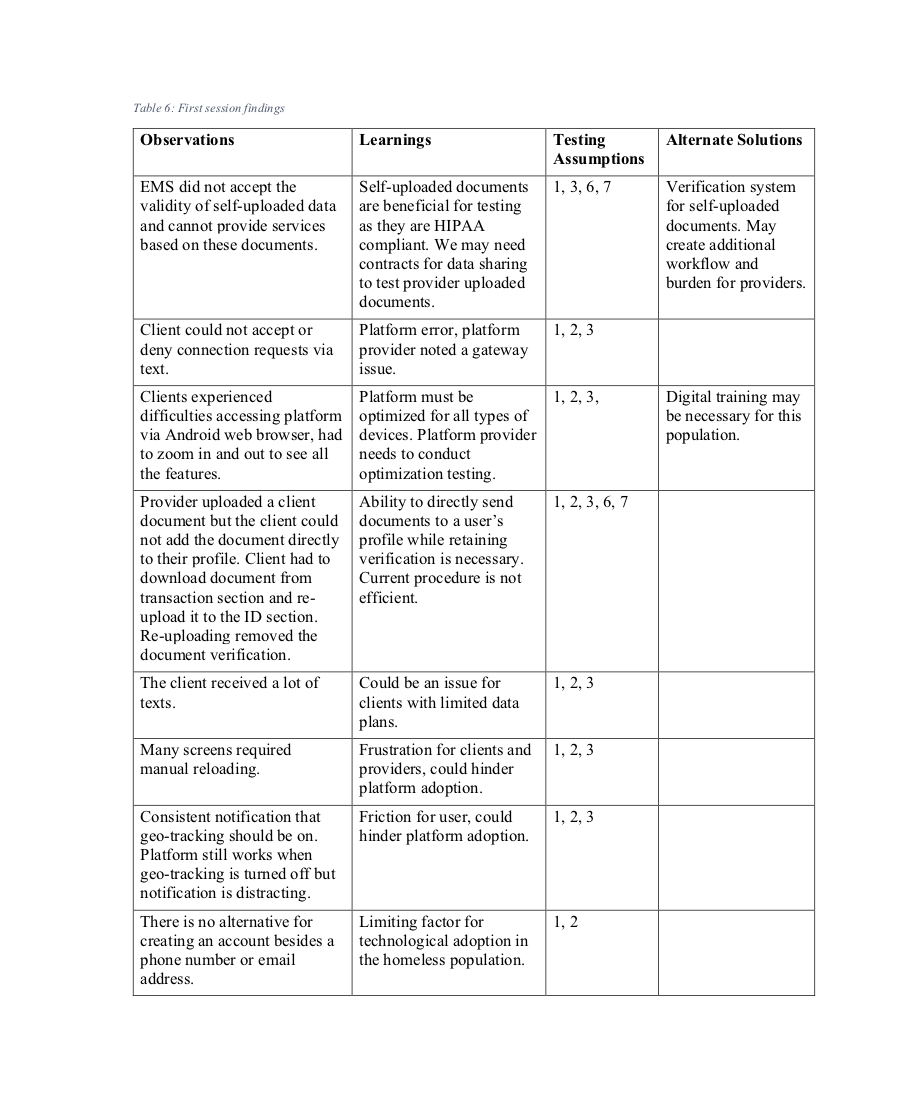

Supplement: Multimedia Appendix 2 [file jmir_v22i6e16887_app2.png]

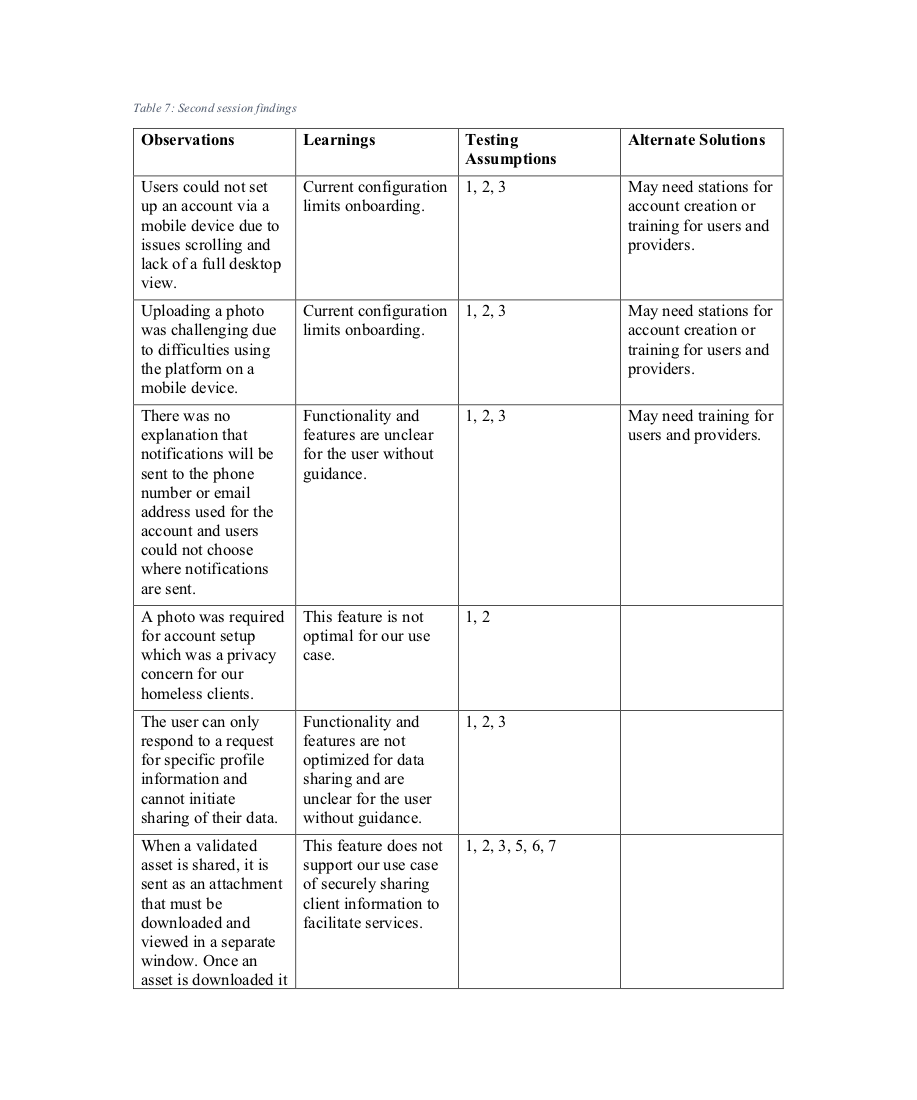

Supplement: Multimedia Appendix 3 [file jmir_v22i6e16887_app3.png]
